# Iron Control in Liquid Effluents: Pseudo-Emulsion Based Hollow Fiber Membrane with Strip Dispersion Technology with Pseudo-Protic Ionic Liquid (RNH_3_^+^HSO_4_^−^) as Mobile Carrier

**DOI:** 10.3390/membranes13080723

**Published:** 2023-08-08

**Authors:** Francisco Jose Alguacil, Jose Ignacio Robla

**Affiliations:** Centro Nacional de Investigaciones Metalurgicas (CSIC), Avda. Gregorio del Amo 8, 28040 Madrid, Spain; jrobla@cenim.csic.es

**Keywords:** iron(III), pseudo-protic ionic liquid, Primene JMT, sulphuric acid, pseudo-emulsion-based hollow fiber strip dispersion

## Abstract

The transport of iron(III) from aqueous solutions through pseudo-emulsion-based hollow fiber with strip dispersion (PEHFSD) was investigated using a microporous hydrophobic hollow fiber membrane module. The pseudo-protic ionic liquid RNH_3_HSO_4_^−^ dissolved in Solvesso 100 was used as the carrier phase. This pseudo-protic ionic liquid was generated by the reaction of the primary amine Primene JMT (RNH_2_) with sulphuric acid. The aqueous feed phase (3000 cm^3^) containing iron(III) was passed through the tube side of the fiber, and the pseudo-emulsion phase of the carrier phase (400 cm^3^) and sulphuric acid (400 cm^3^) were circulated through the shell side in counter-current operational mode, using a single hollow fiber module for non-dispersive extraction and stripping. In the operation, the stripping solution (sulphuric acid) was dispersed into the organic membrane phase in a tank with a mixing arrangement (a four-blade impeller stirrer) designed to provide strip dispersion. This dispersed phase was continuously circulated from the tank to the membrane module in order to provide a constant supply of the organic solution to the fiber pores. Different hydrodynamic and chemical parameters, such as feed (75–400 cm^3^/min) and pseudo-emulsion phases (50–100 cm^3^/min) flows, sulphuric acid concentration in the feed and stripping phases (0.01–0.5 M and 0.5–3 M, respectively), metal concentration (0.01–1 g/L) in the feed phase, and PPILL concentration (0.027–0.81 M) in the carrier phase, were investigated. From the experimental data, different diffusional parameters were estimated, concluding that the resistance due to the feed phase was not the rate-controlling step of the overall iron(III) transport process. It was possible to concentrate iron(III) in the strip phase using this smart PEHFSD technology.

## 1. Introduction

**Figure 1:**
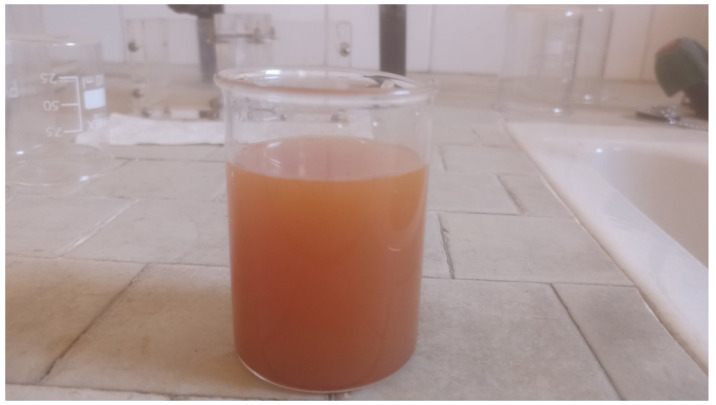
Presence of iron(III) in tap water due to degradation of pipelines (picture Alguacil and Robla).

Iron(III) is an element in close relationship with Mankind, both for natural and anthropogenic causes. This element widely exists in the environment and is considered essential for humans and generally for life however, low or high iron levels in the human organism can be the cause of different diseases. Low iron levels in the body promote anemia, whereas high iron concentrations lead to organ damage, arthritis, liver disease, etc.; thus, high iron(III) concentrations in water are the cause of environmental problems. The presence of iron(III) in waters is accompanied by some characteristics such as odour, taste, and colour (Figure 1); its presence also causes corrosion and staining effects [1,2,3,4].

Also, iron(III) is an often-found constituent in many raw materials; thus, in the production, via Pyrometallurgical or Hydrometallurgical technologies, of other most valuable metals, a step devoted to the removal of this non-profitable iron(III) is very often considered.

In conclusion, extraction or removal of iron(III) from high to trace concentrations plays an important role in many fields, i.e., waste stream treatment, determination of iron in minerals, separation and purification of radionuclide solutions, water treatment, organic synthesis, etc.

It is of the utmost necessity to develop effective strategies for the detection and removal of iron(III) in water environments. These strategies included the use of different separation technologies from precipitation to adsorption, with recent publications considering liquid-liquid extraction using (acidic, solvation, and ionic liquid-based extractants) [5,6,7,8,9], ion-exchange resins (Lewatit TP-208) [2], adsorption (carbon derivatives, zeolites, and microgels) [10,11,12,13], and membrane [4,14] procedures.

In the case of membrane technologies, supported liquid membranes both in flat-sheet or hollow fiber modules are gaining special consideration in the recovery of metals [15,16,17,18]. In the case of hollow fiber membrane processing, one of the more advanced developments is the so-called pseudo-emulsion-based hollow fiber strip dispersion (PEHFSD), which is characterised by its ability to afford high mass transfer rates of solutes and is accompanied by high selectivity by the use of specific carriers or extractants.

Among these extractants, ionic liquids are considered one of the smartest types of reagents to be used in the recovery of metals from different sources. These ionic liquids have gained widespread use due to their properties [19,20]: low volatility and flammability [21,22], high refractive index and thermal stability [23,24], strong conductivity and selectivity [25], and solvation power of both organic and inorganic compounds [26,27]. Mostly due to these properties, this type of reagent is considered a *green solvents*, though their environmental greenness status is also under debate [28,29].

Moreover, ionic liquids can also be divided into some subfamilies [30]: (*i*) aprotic ionic liquids and task-specific ionic liquids, both types being considered fully ionic; (*ii*) protic ionic liquids, generated by the reaction of an acid and a base; and (*iii*) pseudo-protic ionic liquids (PPILs), which are also generated by the reaction of an acid and a base but are sometimes considered not fully ionic. A special case of these (*iii*) types of compounds is the PPILS formed from tertiary amines [31,32], and probably for extension and reactive similarities by primary and secondary amines [33].

The present work presented an investigation about the facilitated transport of iron(III) using a pseudo-protic ionic liquid (RNH_3_^+^HSO_4_^−^), derived from the reaction of the primary amine Primene JMT and sulphuric acid, as a mobile carrier and pseudo-emulsion-based hollow fiber strip dispersion membrane operation. The influence of different variables, such as hydrodynamic conditions and chemical parameters, is considered in order to yield efficient membrane technology.

## 2. Materials and Methods

### 2.1. Materials

Amine Primene JMT (Dow Chem., Midland, MI, USA) and sulphuric acid are the precursors of the pseudo-protic ionic liquid (PPIL) used in this work. Primene JMT is a primary amine having highly branched alkyl chains (C_16–22_), in which the nitrogen atom of the amine group is linked to a tertiary carbon atom. The amine has an average molecular weight of 315 and a density (25 °C) of 0.840 g/cm^3^ [34].

Solvesso 100 is an aromatic (99%) diluent (Exxon Chem Iberia, Madrid, Spain). All other reagents used in the work are of AR grade.

The hollow fiber module device used in the investigation was manufactured by Hoechst Celanese: Liqui-Cel 8 × 28 cm 5PCG-259 contactor and 5PC5-1002 Liqui-Cel laboratory LLE, whose specifications are summarised in Table 1.

### 2.2. Methods

#### 2.2.1. Liquid-Liquid Extraction Experiments (Generation of the PPIL)

To establish conditions for the generation of the pseudo-protic ionic liquid (RNH_3_^+^HSO_4_^−^) used as a carrier in this work, several liquid-liquid extraction experiments were carried out under the following conditions: equal volumes of 25 cm^3^ each containing 1 M sulphuric acid and the amine dissolved in Solvesso 100 were put in thermostatically controlled (25 °C) glass vessels and mechanically shaken via four bladed glass impellers. Equilibrium was reached within a few minutes (less than 5 min). After phase disengagement, the acidic content in the organic phase was analysed by titration in ethalonic medium with standard NaOH solutions, using bromothimol blue as an indicator, whereas the acid content in the corresponding equilibrated aqueous phases was analysed in the same manner. The distribution coefficient of sulphuric acid, defined as the ratio of concentrations of the acid in the organic phase (org) and in the aqueous phase (aq), at equilibrium, was calculated as:

(1) $$D_{H_{2}SO_{4}}=\frac{{\left[H_{2}SO_{4}\right]}_{org}}{{\left[H_{2}SO_{4}\right]}_{aq}}$$

#### 2.2.2. Membrane Operation

The hollow fiber strip dispersion operation comprised a single membrane contactor, one stirred tank containing the pseudo-emulsion phase formed by the PPIL solution (carrier phase) and sulphuric acid solution (stripping phase), and one stirred tank containing the iron(III)-bearing feed phase. The set-up also contained two gear pumps to provide variable flows for both phases and flow meters.

In the operation, the organic solution wet the porous wall of the fiber due to its hydrophobic nature, whereas the interface was maintained at the pore by the application of a higher pressure to the feed phase than to the pseudo-emulsion phase. This differential pressure was set below the breakthrough pressure; in the present investigation, the pressure applied to the feed phase was 0.2 bar higher than the corresponding pressure applied to the pseudo-emulsion phase.

**Figure 2:**
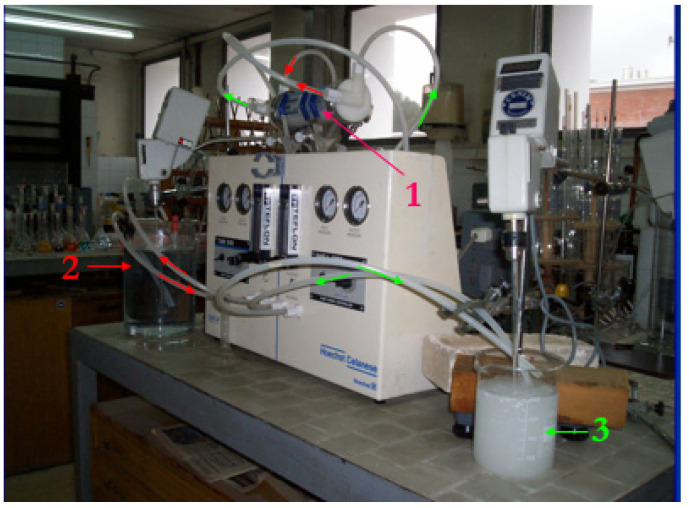
View of the hollow fiber module operating in the recycling mode of the phases. (1) Hollow fiber membrane contactor, (2) feed phase tank, and (3) pseudo-emulsion phase tank. Red colour: feed phase flow. Green colour: pseudo-emulsion phase flow.

A view of the membrane operation using one contactor and in the recirculation mode is shown in Figure 2.

The membrane operation was carried out by passing the iron(III)-bearing feed phase across the tube side of the fibers and the pseudo-emulsion phase through the shell side in counter-current mode. The tank containing the feed phase was stirred in order to homogenise the solution, whereas the stirring of the tank containing the pseudo-emulsion phase was needed in order to maintain the pseudo-emulsion and homogenise it. In addition, the characteristics of the pseudo-emulsion must be such that it must have clear and fast organic and strip-phase disengagement when mixing is stopped; thus, the pseudo-emulsion reservoir tank acts as a mixer-settler.

In the operation, the volume of the pseudo-emulsion phase was 800 cm^3^ (400 cm^3^ each of the organic and stripping solutions), whereas the volume of the feed phase was 3000 cm^3^. At elapsed times, aliquots of the feed and pseudo-emulsion tanks were taken and analysed for iron concentration in the aqueous solutions by conventional atomic absorption spectrometry (Perkin Elmer 1100B spectrophotometer). The permeation coefficient (P) was calculated by the next equation:

(2) $$\ln\frac{{\left[Fe\right]}_{f,t}}{{\left[Fe\right]}_{f,0}}=-\frac{A\cdot P}{V}t$$

where A was the membrane area (1.4 m^2^), V was the volume (3000 cm^3^) of the feed phase, and t was the elapsed time. In the above equation, [Fe]_f,t_ and [Fe]_f,0_ were the iron(III) concentrations in the feed phase at an elapsed time and time zero, respectively.

## 3. Results and Discussion

### 3.1. Generation of the Pseudo-Protic Ionic Liquid (PPIL)

Upon reaction of the amine (RNH_2_) with sulphuric acid, the pseudo-protic ionic liquid was generated according to the next reactions:

(3) $$RNH_{2_{org}}+H_{2}SO_{4_{aq}}\Leftrightarrow {\left(RNH_{3}^{+}\right)}_{2}SO_{4_{org}}^{2-}$$

(4) $${\left(RNH_{3}^{+}\right)}_{2}SO_{4_{org}}^{2-}+H_{2}SO_{4_{aq}}\Leftrightarrow 2RNH_{3}^{+}\cdot HSO_{4_{org}}^{-}$$

Thus, firstly, the amine sulphate (RNH_3_^+^)_2_SO_4_^2−^ was formed (Equation (3)), and in excess of acid, the amine bisulfate RNH_3_^+^HSO_4_^−^ was generated (Equation (4)). The overall reaction being:

(5) $$RNH_{2_{org}}+H_{2}SO_{4_{aq}}\Leftrightarrow RNH_{3}^{+}\cdot HSO_{4_{org}}^{-}$$

To estimate the extraction equilibrium for Equation (5), experiments were carried out mixing organic phases of the amine (0.068–0.54 M) in Solvesso 100 and 1 M sulphuric acid solutions. The results of the acid extraction by the amine were summarised in Table 2, in which the acid extraction into the organic phase was calculated as the acid distribution coefficient (Equation (1)).

In Equation (5), the acid extraction constant (K_ext_) was defined as:

(6) $$K_{ext}=\frac{{\left[RNH_{3}^{+}\cdot HSO_{4}^{-}\right]}_{org}}{{\left[RNH_{2}\right]}_{org}{\left[H_{2}SO_{4}\right]}_{aq}}$$

and taking into consideration Equation (1), taking log, and rearranging, the next expression was obtained:

(7) $$\log D_{H_{2}SO_{4}}=\log K_{ext}+\log{\left[RNH_{2}\right]}_{org}$$

**Figure 3:**
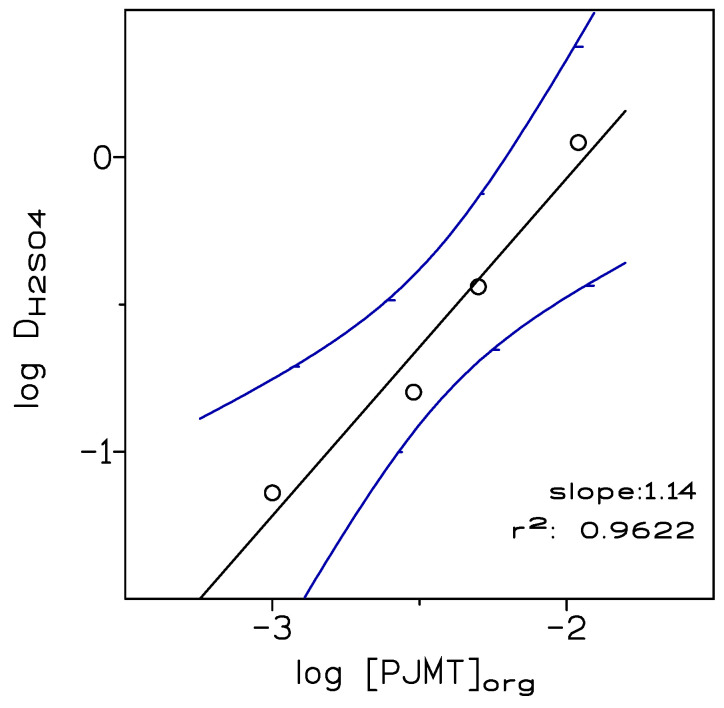
Plot of log D_H2SO4_ versus log [PJMT]_org_. The dotted line shows the 95% confidence interval of the regression line. Experimental conditions as in the text and Table 2.

Thus, a plot of log D_H2SO4_ versus log [RNH_2_]_org_ allowed us to estimate the stoichiometric coefficient (slope) and the value of log Kext (ordinate). Figure 3 showed such a plot, and it can be seen that the slope was close to 1 (as expected from Equation (5), with log K_ext_ 2.22).

The results of Table 2 were numerically treated by a computer programme that minimised the U function, defined as:

(8) $$U=\Sigma {\left(\log D_{\exp}-\log D_{cal}\right)}^{2}$$

where D_exp_ was the experimental sulphuric acid distribution coefficient and D_cal_ was the corresponding one calculated by the programme. The results from these calculations confirmed the formation of the pseudo-protic ionic liquid (RNH_3_^+^·HSO_4_^−^) and the stoichiometry proposed in Equation (5). The value of lo K_ext_ (U = 0.0006) calculated by the programme was 2.25 ± 0.26, which was close to the graphical value obtained from Figure 3.

### 3.2. Hollow Fiber Membrane Experiments

The iron(III) flux (J) through the hollow fiber module was given by the next relationship:

(9) $$J=P\left[{\left[Fe\right]}_{f}-\left(\frac{D_{Fe,st}}{D_{Fe,f}}\right){\left[Fe\right]}_{st}\right]$$

where P was the permeation coefficient, [Fe]_f_ and [Fe]_st_ were the iron concentrations in the feed and in the strip solutions, D_Fe,f_ was the iron distribution coefficient between the membrane and the feed phases, at the reaction equilibrium, at the feed phase-side interface, and D_Fe,st_ was the iron distribution coefficient between the membrane and the strip phases, at the reaction equilibrium, at the strip-side interface. In practise, D_Fe,f_ values were much greater than D_Fe,st_ values, and the second term between the squares in Equation (9) can be neglected in comparison with [Fe]_f_. Thus, the mass balance in the feed solution can be expressed as:

(10) $$-V\frac{d{\left[Fe\right]}_{f}}{dt}=A\cdot P\cdot {\left[Fe\right]}_{f}$$

where V was the feed phase volume, A represented the membrane area, and t was the elapsed time. Integration of the above resulted in Equation (2).

The operation of the hollow fiber module for the removal and concentration of iron(III) using the overall permeation coefficient was based on three mass transfer resistances: (*i*) one occurring in the solution flowing through the tube side of the fiber; (*ii*) a second, corresponding to the diffusion of the iron-PPIL complex across the immobilised liquid membrane located in the fiber pores; and (*iii*) the resistance due to the aqueous interface created on the outside of the hollow fiber.

Thus,

(11) $$\frac{1}{P}=\frac{1}{k_{a}}+\frac{r_{i}}{r_{\ln}}\frac{1}{P_{m}}+\frac{r_{i}}{r_{o}}\frac{1}{k_{o}}$$

where k_a_ and k_o_ were the mass transfer coefficients corresponding to the inner and outer aqueous boundary layers, and r_ln_ was the hollow fiber log mean radius. The membrane permeability (P_m_) was related to the distribution coefficient by the next equation:

(12) $$P_{m}=D_{Fe,f}k_{m}$$

where k_m_ represents the membrane mass transfer coefficient. Inserting Equation (12) into Equation (11) resulted in:

(13) $$\frac{1}{P}=\frac{1}{k_{a}}+\frac{r_{i}}{r_{\ln}}\frac{1}{D_{Fe,f}k_{m}}+\frac{r_{i}}{r_{o}}\frac{1}{k_{o}}$$

When the reaction was instantaneous on the stripping side, the contribution of the outer aqueous phase can be removed from Equation (13), and the overall permeability coefficient is expressed as:

(14) $$\frac{1}{P}=\frac{1}{k_{a}}+\frac{r_{i}}{r_{\ln}}\frac{1}{D_{Fe,f}k_{m}}$$

#### 3.2.1. Influence of the Feed Phase Flow on Iron(III) Permeation

The influence of this variable on iron(III) permeation was first investigated by using a feed phase containing 0.01 g/L Fe(III) and 0.1 M sulphuric acid medium and a pseudo-emulsion phase containing 0.27 M PPIL in Solvesso 100 as organic solution and 3 M sulphuric acid as stripping solution.

The results of this set of experiments are shown in Table 3. It can be seen that the permeation coefficient increased with the increase in feed phase flow up to 300 cm^3^/min and decreased at a higher feed phase flow. In a transport process across a liquid membrane, two types of diffusional resistances can be considered: (*i*) the resistance due to the feed phase boundary layer and (*ii*) the resistance associated with the membrane support. It is often found that the magnitude of the first competes with the value of the support resistance [35]. The experimental results shown in Table 4 indicated that at 300 cm^3^/min, the feed boundary layer was at a minimum and the feed phase resistance to mass transfer was minimised, thus, the diffusion contribution of the aqueous species to the mass transfer phenomena can be considered constant [36].

The decrease of the permeation coefficient value at flows exceeding 300 cm^3^/min can be explained in terms of (*i*) the increase of the turbulence in the feed phase, which forced organic solution out of the membrane pores; (*ii*) the lower residence time of the feed phase in the module as a consequence of increasing the flow; and (*iii*) the formation of an emulsion along the lumen side also due to the increase of the flow [37].

#### 3.2.2. Influence of the Pseudo-Emulsion Phase Flow on Iron(III) Permeation

Using the same feed and pseudo-emulsion phases as in the previous subsection and a feed phase flow of 300 cm^3^/min, a series of experiments were conducted to investigate the influence of the pseudo-emulsion phase flow on iron(III) permeation. These experiments showed that in the 50–100 cm^3^/min flow range, the variation of these flows had a negligible influence on the removal of iron(III) from the feed phase.

#### 3.2.3. Influence of the Strip Solution Composition on Iron(III) Permeation

The permeation of iron(III) under different strip solutions was also investigated. In these experiments, the feed phase contained 1 g/L Fe(III) in 0.1 M sulphuric acid medium, whereas the pseudo-emulsion phase was contained and an organic solution of 0.14 M PPIL in Solvesso 100 and 0.5–3 M sulphuric acid solution were used as strippants.

The values of the iron(III) permeation coefficients derived from the experimentation are shown in Table 4. These values showed that the removal of iron(III) from the feed phase increased (higher permeation coefficient) with the increase in sulphuric acid concentration in the stripping solution. In addition, metal recovery in these stripping solutions increased with the increase of in acid concentration; this result was especially noted in the case of using 0.5 M acid versus 1.5 or 3 M sulphuric acid solutions in the strip solution.

#### 3.2.4. Influence of the Sulphuric Acid Concentration in the Feed Phase on Iron(III) Permeation

The variation of the sulphuric acid concentration in the feed phase on iron(III) permeation was also investigated by using feed phases of 0.01 g/L Fe(III) and varying acid concentrations and pseudo-emulsion phases containing 0.14 M PPIL in Solvesso 100 and 3 M sulphuric acid.

**Figure 4:**
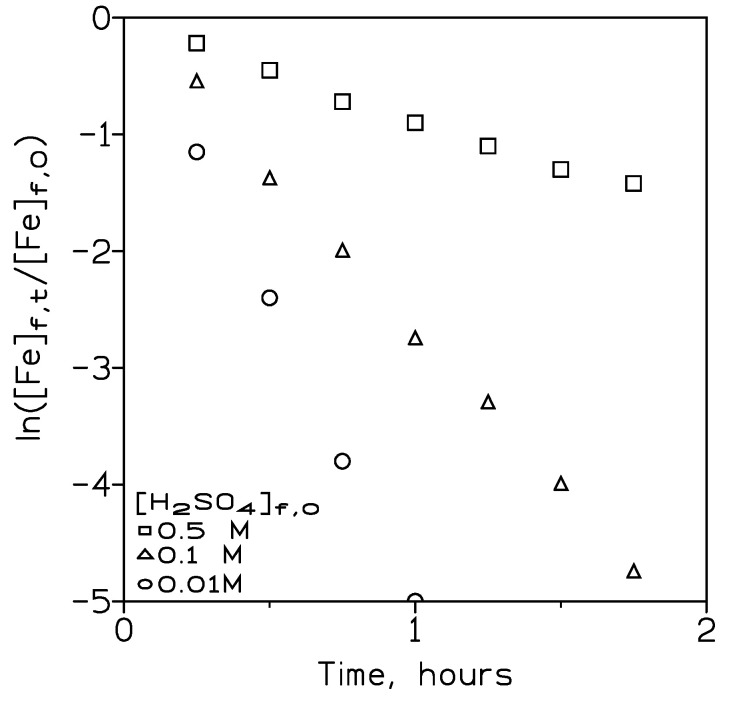
Plot of ln([Fe]_f,t_/[Fe]_f,0_) versus time at various initial sulphuric acid concentrations in the feed phase. Feed phase flow: 300 cm^3^/min. Pseudo-emulsion flow: 100 cm^3^/min. Temperature: 20 °C.

These results were presented in Figure 4, plotting ln ([Fe]_f,t_/[Fe]_f,0_) versus time at the various acid concentrations in the feed phase. It was concluded that the variation of the acid concentration in the feed phase had a key influence on the removal of iron(III) from this phase since the permeation coefficient value decreased from 1.8·10^−2^ cm/min using feed phases containing 10^−2^ M acid to 3.0·10^−3^ cm/min when the feed phase contained 0.5 M sulphuric acid.

#### 3.2.5. Influence of the Initial Iron(III) Concentration in the Feed Phase on Metal Extraction

Using a pseudo-emulsion phase of 0.14 M PPIL in Solvesso 100 and 3 M sulphuric acid as a stripping solution, the variation of the initial metal concentration in the feed phase on the iron(III) permeation coefficient was investigated. In these experiments, the feed phase contained 0.01–1 g/L Fe(III) in 0.1 M sulphuric acid medium, and flows of 300 cm^3^/min and 100 cm^3^/min were used in the feed and pseudo-emulsion phases, respectively.

**Figure 5:**
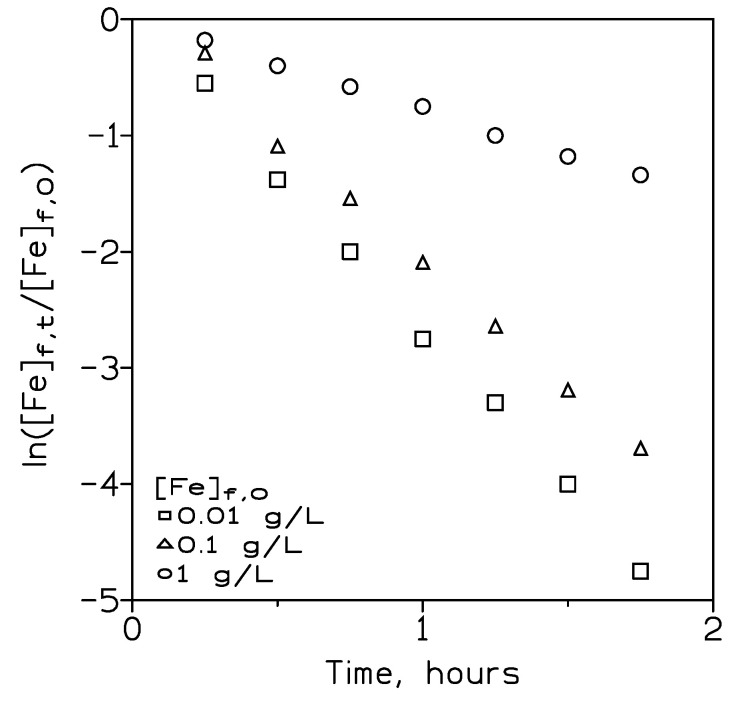
Iron(III) transport at various initial metal concentrations in the feed phase. Temperature: 20 °C.

The results of these experiments (Figure 5) indicated that the increase of the metal concentration in the feed phase decreased iron(III) permeation. These results can be explained in terms of the fact that as the iron(III) concentration in the feed phase increased, the organic phase immobilised in the fiber pores became saturated with the iron-PPIL complex. Further, this complex diffused at a slow rate into the bulk of the organic solution, which resulted in a decrease in the mass transfer in the organic solution. However, this slow transfer can be remedied either by increasing the membrane surface or running the transport operation for longer periods of time.

The initial metal flux (J) can be defined by the relationship:

(15) $$J=P\cdot {\left[Fe\right]}_{f,0}$$

Table 5 shows the flux values corresponding to the various initial iron(III) concentrations used in this work.

These results showed that, as somewhat expected from Equation (15), the iron flux increased with the increase in the initial metal concentration in the feed phase [38].

#### 3.2.6. Influence of the Carrier Concentration on Iron(III) Permeation

It was obvious that the presence of the carrier in the pseudo-emulsion phase was another key factor in achieving good metal permeation across the hollow fiber membrane and, thus, a convenient removal of this undesirable solute in a given feed phase. To investigate the effect of varying the pseudo-protic ionic liquid (carrier) concentration in the organic phase on iron(III) permeation, experiments were conducted using feed phases of 0.01 g/L Fe(III) in 0.1 M sulphuric acid medium and pseudo-emulsion phases of various carrier concentrations in Solvesso 100 and 3 M sulphuric acid concentrations.

**Figure 6:**
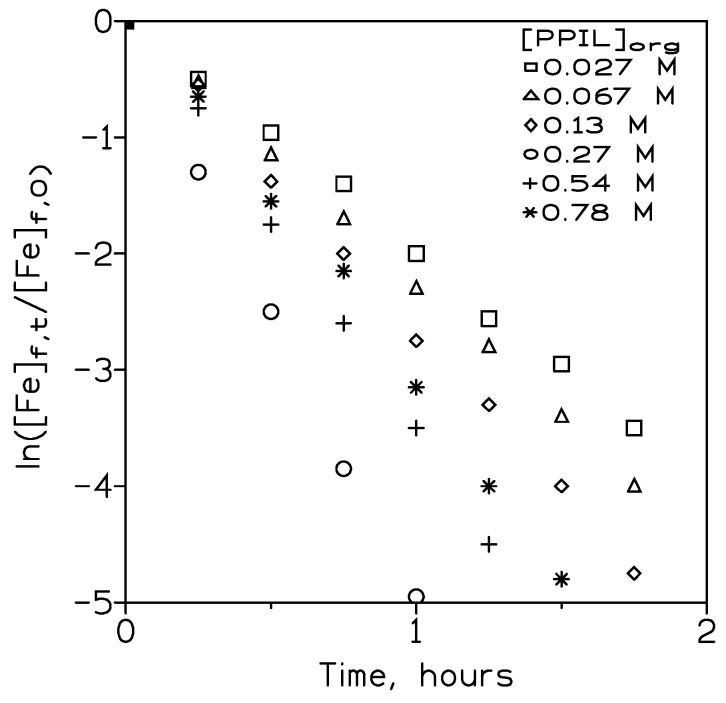
Variation with time of ln([Fe]_f,t_/[Fe]_f,0_) at various PPIL concentrations. Feed phase flow: 300 cm^3^/min. Pseudo-emulsion flow: 100 cm^3^/min. Temperature: 20 °C.

The results derived from these series of experiments were shown in Figure 6, which showed that the removal of iron(III) from the feed phase increased with the increase of the carrier concentration from 0.027 M to 0.27 M; these results indicated that in this range of concentrations, iron(III) transport was governed by membrane diffusion, though in the maximum transport or permeability region, diffusion in the membrane fibers was negligible and the transport rate was therefore limited by diffusion through the boundary film of the aqueous solution on the feed side of the fibers. However, at carrier concentrations higher than 0.27 M, iron(III) permeation decreased, probably as a consequence of an increase in the organic phase viscosity due to the increase in the carrier concentration in the organic solution, which resulted in a decrease in iron(III) transport across the fiber pores [39].

Table 6 showed the permeation coefficient values at these various PPIL concentrations. This table also showed the percentage of iron(III) recovered in the stripping solution. It can be seen that the percentage of iron(III) recovered in the strip solution was in excess of 80%.

#### 3.2.7. Estimation of Diffusional Parameters

It can be assumed that the PPIL concentration in the membrane module was constant; under this assumption, the apparent diffusion coefficient for iron(III) can be calculated by the next expression [40]:

(16) $$D_{0}^{a}=\frac{J\cdot d_{org}}{\left[PPIL_{org}\right]}$$

being J the metal flux (Table 4), d_org_ the fiber thickness (Table 1), and using a PPIL concentration of 0.27 M (concentration at which maximum iron(III) permeation was obtained), the value of this apparent diffusion coefficient was calculated as 1.9·10^−8^ cm^2^/min.

An estimation of the membrane mass transfer coefficient (k_m_) can be performed using the next relationship [41]:

(17) $$k_{m}=\frac{D_{org}\cdot \varepsilon }{\tau \left[\frac{d_{o}-d_{i}}{2}\right]}$$

In the above expression, D_org_ was the diffusion coefficient of the metal-carrier species in the fibers, with an average value of 6·10^−5^ cm^2^/min [42], ε was the fiber porosity, τ was the fiber tortuosity, and d_o_ and d_i_ were the values of the outer and inner fiber diameters, respectively (values of ε, τ, d_o_, and d_i_ were given in Table 1). In the present investigation, the value of k_m_ was estimated at 3.3·10^−5^ cm/min.

The membrane mass transfer coefficient did not depend on the hydrodynamic conditions applied to a system; it was only related to the fiber properties and the diffusion coefficients of the extracted complex in the organic solution filling the fiber pores.

The effective diffusion coefficient (D_eff_) of iron(III)-PPIL complex across the organic membrane phase was also determined. This diffusion coefficient for the solute in the immobilised organic liquid membrane was calculated as [43,44]:

(18) $$D_{eff}=k_{m}\cdot d_{org}\cdot \tau$$

where d_org_ is the hollow fiber thickness (Table 1). Thus, the value of D_eff_ for the present system was calculated to be near 2.0·10^−3^ cm^2^/min.

It was previously mentioned (Section 3.2.) that the distribution coefficient in the feed phase (D_Fe,f_) was normally much greater than the distribution coefficient in the stripping solution (D_Fe,st_), and it was also often considered that the stripping reaction was instantaneous; thus, the mass transfer resistance for the strip solution was negligible if compared with the overall mass transfer resistance. The individual feed mass transfer coefficient (k_a_) was dependent on the mean flow velocity of the feed phase (u_a_) [45]:

(19) ka=1.5Daqdidi2·uaDaq·L13

where D_aq_ was the diffusion coefficient or iron species in the feed phase (averaging 6·10^−4^ cm^2^/min [46]), d_i_ the inner diameter of the fibers, and L was the fiber length (Table 1). Thus, at 300 cm^3^/min, the value of k_a_ can be calculated as 6.1·10^−2^ cm/min.

Equations (13) and (14) showed that the overall resistance was the sum of the values of the individual resistances, and the results shown in Table 6 indicated the overall resistance values were in the 55–155 min/cm range, whereas the value of the resistance due to the feed phase (R_a_ = 1/k_a_) was 16 min/cm. The fractional resistance due to the feed phase (R_a_^0^) to the overall process (R) can be calculated as:

(20) $$R_{a}^{0}=\frac{R_{a}}{R}\cdot 100$$

Under the present experimental conditions, the value of Ra^0^ was 17%, this value clearly indicated that this step was not rate-controlling the overall iron(III) transport process.

## 4. Conclusions

Hollow fiber membrane, in the strip dispersion operational form, investigations were carried out in a single membrane module for simultaneous extraction and stripping in counter-current mode. From the results derived from this investigation, it can be concluded that the experimental conditions: (*i*) using a pseudo-emulsion phase consisting of a mixture of 0.27 M RNH_3_^+^HSO_4_^−^ (pseudo-protic ionic liquid derived from the reaction of the primary amine Primene JMT and sulphuric acid) in Solvesso 100 and 3 M sulphuric acid, and maintaining flows of 300 cm^3^/min and 100 cm^3^/min for feed and pseudo-emulsion phases, respectively, were suitable for the efficient removal and concentration of iron(III) under optimum conditions.

From the experimental data, several diffusional parameters were calculated, and it was shown that iron(III) transport rate was mostly controlled by membrane diffusion. Under proper operation, the stability of PEHFSD was found to be good. This type of membrane operation presented a promising alternative to conventional separation methodologies and should increase its interest for the potential removal of iron(III) (and other undesirable solutes) as well as the recovery of valuable metals from liquid effluents, especially when the concentrations of these solutes are low.

## Figures and Tables

**Table 1:** Specifications of the hollow fiber module.

Contactor length	28 cm
Contactor diameter	8 cm
Active area (A)	1.4 m^2^
Number of fibers (n)	10,000
Fiber internal diameter (di)	24·10^−3^ cm
Fiber outer diameter (do)	30·10^−3^ cm
Fiber wall thickness (dorg)	3.0·10^−2^ cm
Fiber length (L)	15 cm
Porosity (ε)	30%
Tortuosity (τ)	3
Pore size	3.0·10^−6^ cm
Polymeric material	3.0·10^−6^ cm

**Table 2:** Generation of the pseudo-protic ionic liquid (Equation (5)).

[Amine], M	D_H2SO4_
0.068	0.072
0.14	0.16
0.27	0.36
0.54	1.1

Temperature: 20 °C. Equilibration time: 10 min. O/A ratio: 1.

**Table 3:** Overall permeation coefficients at various feed phase flows.

Feed Flow, cm^3^/min	P, cm/min
75	2.1·10^–3^
150	4.1·10^−3^
300	6.9·10^−3^
400	4.7·10^−3^

Pseudo-emulsion phase flow: 100 cm^3^/min. Temperature: 20 °C.

**Table 4:** Influence of the sulphuric acid concentration in the strip solution on iron(III) permeation.

[H_2_SO_4_], M	P, cm/min	[Fe]_st_, g/L	^a^ % R_st_
0.5	9.9·10^−4^	1.4	50
1.5	1.5·10^−3^	2.9	72
3	2.8·10^−3^	4.2	75

Feed phase flow: 300 cm^3^/min. Pseudo-emulsion phase flow: 100 cm^3^/min. Temperature: 20 °C. ^a^ After 105 min.

**Table 5:** Variation of iron(III) permeation coefficient and initial flux at various initial metal concentrations in the feed phase.

[Fe]_f,0_, g/L	P, cm/min	J, mol/cm^2^·min	[Fe]_st_, g/L	^a^ % R_st_
0.01	9.6·10^−3^	1.7·10^−9^	0.06	84
0.1	7.5·10^−3^	1.3·10^−8^	0.45	80
1	2.8·10^−3^	5.0·10^−8^	4.2	75

Experimental conditions as in Figure 5. ^a^ After 105 min.

**Table 6:** Iron(III) permeation coefficients at various carrier concentrations in the organic solution.

[PPIL], M	P, cm/min	^a^ % R_st_
0.027	6.6·10^−3^	80
0.068	8.1·10^−3^	82
0.14	9.6·10^−3^	84
0.27	1.8·10^−2^	84
0.54	1.3·10^−2^	85
0.81	1.1·10^−2^	83

Experimental conditions as in Figure 6. Temperature: 20 °C. ^a^ After 105 min.

## Data Availability

Not applicable.
